# Multiple functions of Na/K-ATPase in dopamine-induced salivation of the Blacklegged tick, *Ixodes scapularis*

**DOI:** 10.1038/srep21047

**Published:** 2016-02-10

**Authors:** Donghun Kim, Joshua Urban, Daniel L. Boyle, Yoonseong Park

**Affiliations:** 1 Department of Entomology, Kansas State University, 123 Waters Hall, Manhattan, KS 66506, USA; 2 Division of Biology, Microscopy Facility, Kansas State University, Ackert Hall, Manhattan, Kansas 66506, USA

## Abstract

Control of salivary secretion in ticks involves autocrine dopamine activating two dopamine receptors: D1 and Invertebrate-specific D1-like dopamine receptors. In this study, we investigated Na/K-ATPase as an important component of the secretory process. Immunoreactivity for Na/K-ATPase revealed basal infolding of lamellate cells in type-I, abluminal interstitial (epithelial) cells in type-II, and labyrinth-like infolding structures opening towards the lumen in type-III acini. Ouabain (10 μmol l^−1^), a specific inhibitor of Na/K-ATPase, abolished dopamine-induced salivary secretion by suppressing fluid transport in type III acini. At 1 μmol l^−1^, ouabain, the secreted saliva was hyperosmotic. This suggests that ouabain also inhibits an ion resorptive function of Na/K-ATPase in the type I acini. Dopamine/ouabain were not involved in activation of protein secretion, while dopamine-induced saliva contained constitutively basal level of protein. We hypothesize that the dopamine-dependent primary saliva formation, mediated by Na/K-ATPase in type III and type II acini, is followed by a dopamine-independent resorptive function of Na/K-ATPase in type I acini located in the proximal end of the salivary duct.

Ticks are hematophagous arthropod vectors that transmit various disease-causing pathogens to both humans and animals throughout the world. Across the United States, the most commonly reported vector-borne disease is Lyme disease, transmitted by the blacklegged tick, *Ixodes scapularis Say*. Ticks can feed on their host for up to 2 weeks before detaching during which they increase their body weight up to 100 times^1^. The salivary glands (SG) of ticks perform crucial functions before and during this period.

At least three different types of acini of salivary glands are distinguished for the morphs and the functions^2^,^3^,^4^,^5^,^6^,^7^. Type I acini function for absorption of water vapor from the atmosphere by using a hygroscopic saliva in the dehydrated off-host stage^7^,^8^. The salivary secretion at the very early stage of feeding contains immunosuppressive and other bioactive components that promote the successful establishment of the feeding lesion^9^,^10^,^11^,^12^; these are secreted by the granular cells of the type II and type III acini. Another important function of the tick SG is the excretion of excess ions and water from the blood meal (mainly in type III acini). This process concentrates the nutrients needed for growth (larvae and nymphs) and egg production (adult female).

Among the pharmacological reagents known to affect SG secretion, dopamine (DA) and the ergot alkaloids, ergonovine and ergotamine, are the most potent in triggering salivary fluid secretion^13^,^14^,^15^,^16^,^17^,^18^,^19^,^20^. Pilocarpine (PC; a muscarinic cholinergic receptor agonist) stimulates secretion indirectly via the synganglion (CNS of the tick)^15^,^21^. DA produced by the SG^22^,^23^ also affects the SGs via two distinct DA receptors: a dopamine receptor (D1)^24^ and Invertebrate-specific D1-like dopamine receptor (InvD1L)^25^; these receptors mediate distinct physiological actions^26^: influx of water and solutes in types II and III acini, and contraction of myoepithelial cells in types II and III acini, respectively.

The intracellular signaling pathways for DA are only partially known. Na/K-ATPase plays a role in SG function as evidenced by ouabain’s ability to abolish DA-induced salivary secretion^16^,^27^,^28^. Moreover, silencing of the Na/K-ATPase gene via RNA interference demonstrated the importance of Na/K-ATPase to blood feeding and oviposition^29^. An ultrastructural study of type I acini visualized rich Na/K-ATPase-like reactions by the use of a phosphatase cytochemical method^30^.

In this study, we further characterize the roles of Na/K-ATPase in salivary secretion. The effects of ouabain on ion compositions and protein secretion in DA-induced salivation were examined. We propose that there may be distinct physiological roles of Na/K-ATPase for the absorptive and the secretory functions of acini types I and III, respectively.

## Results and Discussion

### Molecular characterization of Na/K-ATPase: two alternatively spliced isoforms, expression patterns, and immunolocalization in the salivary glands

**Figure 1:**
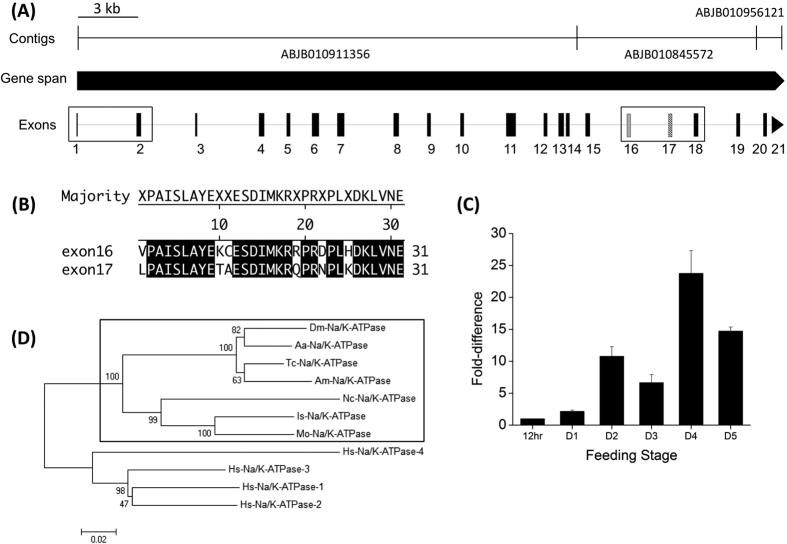
Gene structure, phylogeny, and expression profiles of *I. scapularis* Na/K-ATPase. (**A**) Gene structure of Na/K-ATPase (ISCW002538) including 21 exons. The two boxes indicate corrected regions of the ISCW002538 as determined by manual annotation and experimental confirmation in the present study. The region of exon 1 and exon 2 (exon1 of ISCW002538) is in the first box. The two mutually alternative exons, exons 16 and 17 are in the second box. (**B**) Amino acid sequence alignment of exon16 and exon17. Black shades indicate identical amino acid residues. (**C**) Stage-specific expression profile of Na/K-ATPase, from 12 hrs to 5-days after the onset of feeding. (**D**) Phylogenetic relationship of Na/K-ATPase including *I. scapularis* Na/K-ATPase. The numbers at nodes represent percentage support from 1,000 bootstrap replicates. GenBank Accession numbers: *Metaseiulus occidentali* (XP_003737553.1), *Neoseiulus cucumeris* (AGQ56700.1), *Drosophila melanogaster* (AAF55825.3), *Tribolium castaneum* (XP_008196418.1), *Apis mellifera* (XP_006564225.1), *Aedes aegypti* (XP_001662217.1), and four genes of *Homo sapiens* (NP_000692.2, NP_000693.1, NP_689509.1, and NP_653300.2).

Computational annotation of the gene encoding Na/K-ATPase (ISCW002538) was found in Vectorbase (https://www.vectorbase.org). The gene spans a 37,229 bp long genomic region, consisting of three contigs in the assembled genome sequence; ABJB010911356, ABJB010845527, and ABJB010956121. However, the ISCW002538 prediction lacked a 5′ terminal region, including the untranslated region (UTR) and the start codon. After manual annotation, followed by confirmation via reverse transcriptase (RT)-PCR, we identified an additional 5′ exon (exon 1 in Fig. 1A) linked to the second exon, which is also corrected for 9 bp shorter than the previous ISCW002538 prediction (exon 2 in Fig. 1A). We also identified an alternatively spliced exon, exon 16 (Fig. 1A), which encodes 31 amino acid residues that are homologous to exon 17 (Fig. 1B). Based on RT-PCR results using exon specific primers for exons 16 and 17, we found that these exons are utilized in a mutually alternative manner (one or the other, but not together). Transcripts containing exon 17 appear to be more abundant in the salivary glands, as is evidenced by relative band strengths in the electrophoresis of RT-PCR products (see Supplementary Fig. S1). In addition, exon 18 is 136 bp longer in the 5′ direction than is currently stated in the ISCW002538 prediction. The corrected sequence for Na/K-ATPase was submitted to GenBank with the accession number KU560919 and KU56020. Na/K-ATPase transcript levels were investigated in the SG of females from different feeding stages via quantitative real time RT-PCR (qRT-PCR), revealing a dramatic increase at the onset of feeding, reaching its peak (23-fold increase from the day 1 feeding) at day 4 of feeding, approximately two days before the rapid engorgement phase (Fig. 1C). Phylogenetic analysis using the neighbor joining method showed that the single copy Na/K-ATPases in arthropods are all clustered in a monophyletic group (Fig. 1D).

**Figure 2:**
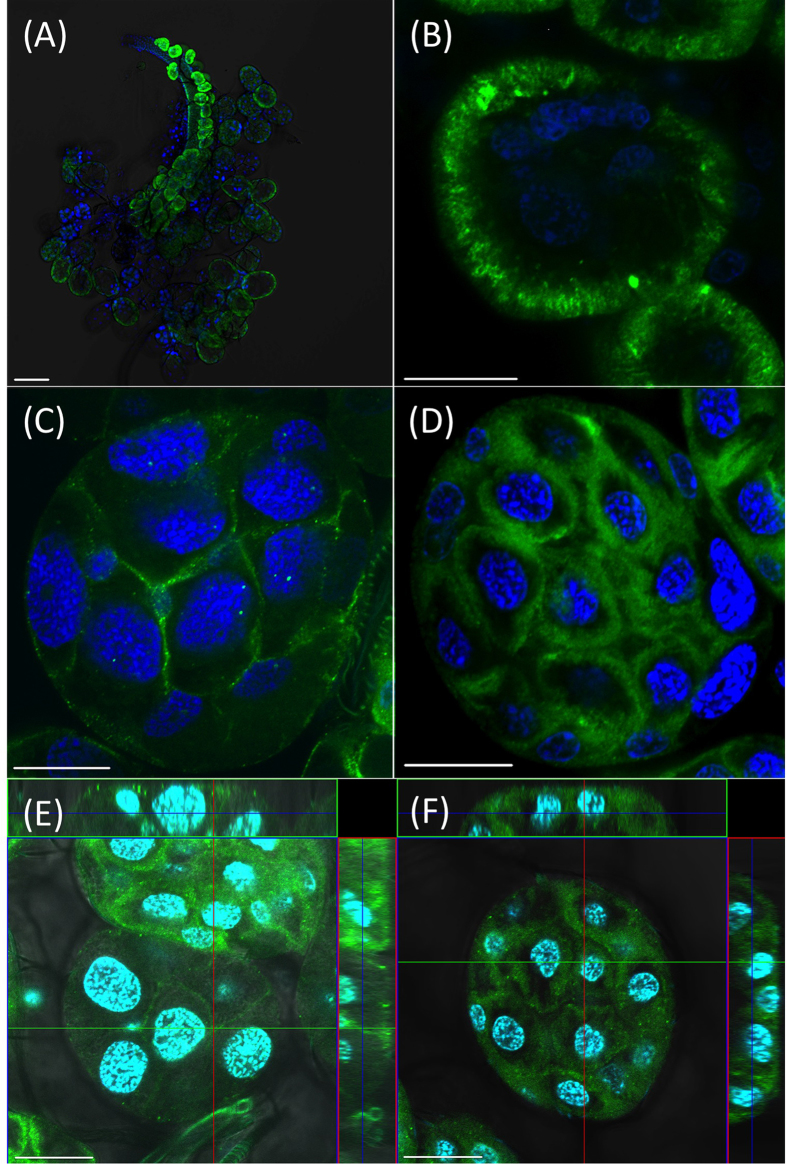
Na/K-ATPase immunoreactivity in three types of salivary gland acini from partially engorged female ticks. (**A**) Overview of salivary glands at low magnification. (**B**) Type I acini. (**C**,**E**) Type II acini. (**D**,**F**) Type III acini. (**E**,**F**) Orthoview of type II and type III acini. Positive staining (green) was detected on the epithelial cells of all types of acini from the salivary glands. The blue color shows nuclei stained with DAPI. Scale bars are for 100 μm (**A**) and 20 μm (**B**–**F**).

In order to localize Na/K-ATPase in tick SG, we used a mouse monoclonal antibody (a5). Na/K-ATPase immunoreactivity was present in the three types of acini (Fig. 2A): lamellate cells in type I (Fig. 2B), abluminal interstitial cells^4^ in type II (Fig. 2C,E), and f-cells^4^ in type III (Fig. 2D,F).

In type I acini (Fig. 2B), immunoreactivity reveals a vertical pattern toward the basolateral surface, as found in a previous study^30^. The immunoreactive abluminal epithelial cells containing small nuclei in type II acini were located between granular cells (Fig. 2E). In type III, the staining pattern was found broadly toward the periphery of each epithelial cell. This subcellular region is likely the place where extensive membrane folding forms a labyrinth, opening towards the lumen, as previously described in the ultrastructure^4^ (Fig. 2F).

### Inhibitory effect of ouabain on dopamine-induced salivary excretion

**Figure 3:**
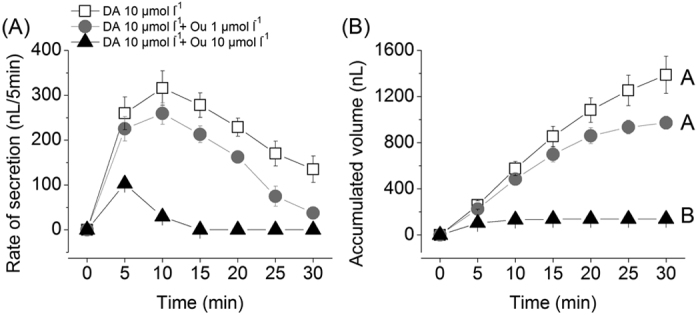
Secretory activities of isolated salivary glands induced by dopamine and ouabain treatments. (**A**) The salivary secretion pattern observed over a 30-minute time period following treatments (**B**) Accumulated saliva volume over a 30-minute time period after treatments. The symbols indicate averages with standard error of the mean (s.e.m.) of three replicates. The data were analyzed by an ANOVA-Tukey-Kramer HSD test (p = 0.05). Different letters indicate significant differences.

A Modified Ramsay’s assay^31^, measuring the amount of salivary secretion from isolated salivary glands, was performed to understand the overall impact of ouabain, a Na/K-ATPase inhibitor, on salivary gland physiology. The rate of DA-induced salivary fluid secretion increased rapidly during the initial 10 minutes, and then waned (Fig. 3A). At 1 and 10 μmol l^−1^ respectively, ouabain inhibited DA-induced secretion by 26% and 90% respectively (Fig. 3) in comparison between the total volumes of secreted saliva for 30 minutes.

**Figure 4:**
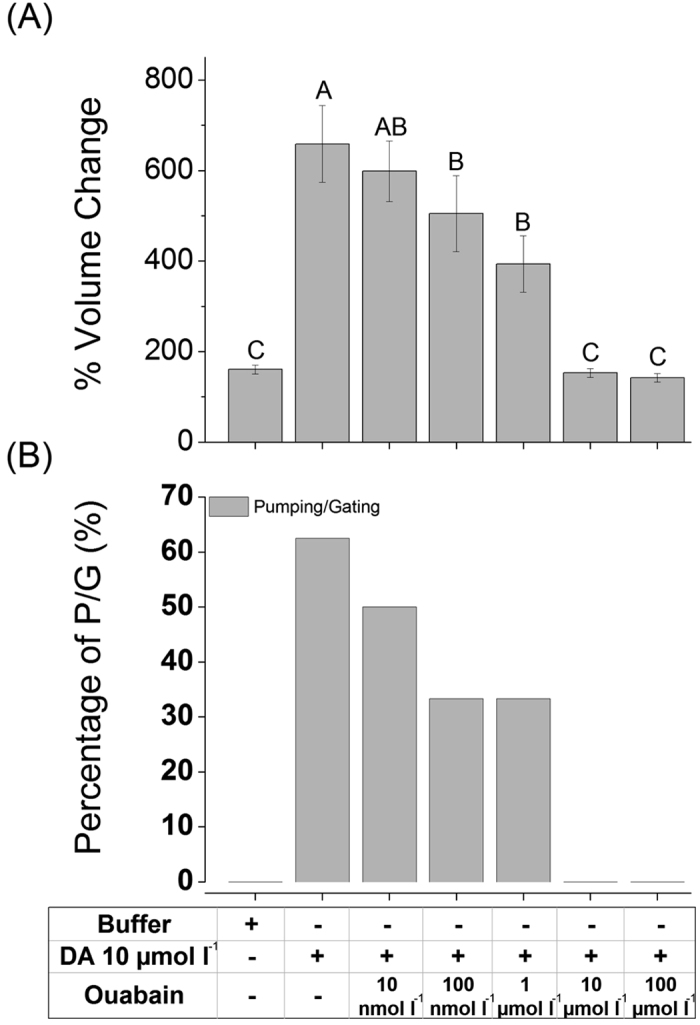
Effect of ouabain on dopamine-mediated fluid influx in type III acini. (**A**) Percentage increase in acinar volume after various combinatory treatments. (**B**) Percentage of acini observed for pumping/gating in each treatment. The data in A are the averages with standard error of mean (s.e.m.) for at least three biological replicates. The data in B are frequencies in observations of more than six acini for each data point. The data were analyzed by an ANOVA-Tukey-Kramer HSD test (p = 0.05). Different letters indicate significant differences.

Next, we employed assay method using microscopic observation of individual acini following treatments in order to the roles of Na/K-ATPase. As previously reported^26^, an influx of water/solutes into type III acini is evidenced by increasing luminal volume. Additionally, dopamine also induced acini pumping/gating for the discharging of saliva via the acinar duct, as described in a previous study^26^. Using a fixed dose of DA (10 μmol l^−1^), we tested the effects of ouabain on acini luminar volume. Ouabain showed dose-dependent suppression of dopamine induced inward fluid transport (Fig. 4A). The percentage volume change of each acinus’ lumen (i.e. water-solute influx) was decreased with increasing doses of ouabain up to 1 μmol l^−1^ (Fig. 4A). At ouabain levels at and above 10 μmol l^−1^, acini dynamics were completely abolished (Fig. 4).

Although earlier studies reported the inhibitory effect of ouabain on catecholamine-induced salivary secretion and on ATPase activity in the salivary glands^16^,^28^,^32^,^33^, here we show that ouabain inhibits water/solute influx into the acinar lumen. The reduced pumping/gating is likely a consequence of this reduced influx. Co-existence of Na/K-ATPase and D1 receptor in the epithelial cells of type II and III acini (this study and Simo *et al.*^24^) suggests that the target of the D1 receptor-mediated intracellular signaling for salivary secretion includes Na/K-ATPase.

DA-induced salivary secretion is orchestrated by two distinct physiological mechanisms^26^: water/solute influx into the lumen via D1 DA-receptor (D1) and discharging the fluid by pumping/gating via InvD1L DA-receptor. This study demonstrates that DA-induced water/solute influx was completely inhibited by ouabain at levels above 10 μmol l^−1^ (Fig. 4A).

### Hyperosmolar dopamine-induced salivary secretion by ouabain

**Figure 5:**
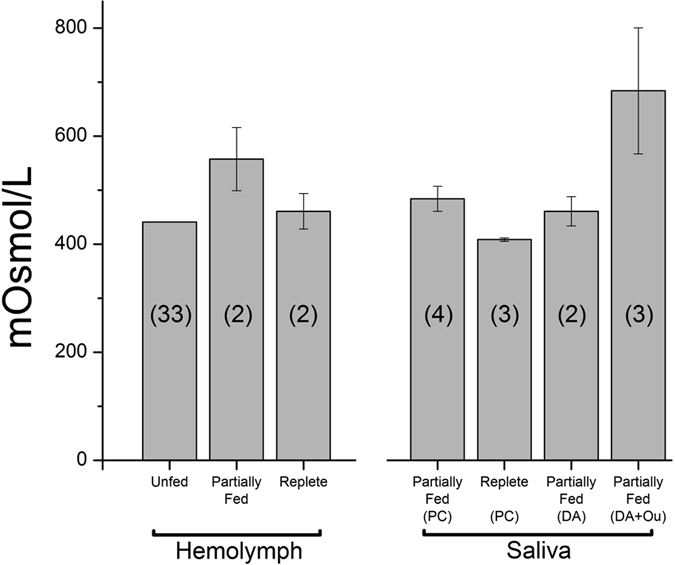
Osmotic pressure of hemolymph and saliva secreted by pilocarpine or dopamine treated ticks. Hemolymph was collected from three different feeding stages: unfed, partially-fed, and replete. Numbers in each bar indicate total number of utilized ticks. The data are the averages with standard error of mean (s.e.m.) for at least two biological replicates. For the collection of saliva, 2 μl of pilocarpine was injected into ticks (partially fed and replete ticks). Saliva collected from dopamine only and dopamine with ouabain were from isolated salivary glands in via the modified Ramsay’s assay. DA: dopamine (10 μmol l^−1^), Ou: ouabain (1 μmol l^−1^), and PC: pilocarpine (10 mg ml^−1^).

We were interested in the effects of dopamine/ouabain on ion concentrations of the salivary secretion. A pre-treatment baseline was established by measuring the osmotic pressures of hemolymph and saliva in adult *I. scapularis* females. Hemolymph osmotic pressure in adult *I. scapularis* females were 441, 557 ± 59, and 461 ± 33 mOsmol/L (±s.e.m.) for unfed, partially fed (11–21 mg), and replete stages, respectively (Fig. 5). These values are similar to those observed in other tick species^7^,^14^,^30^,^34^,^35^.

**Figure 6:**
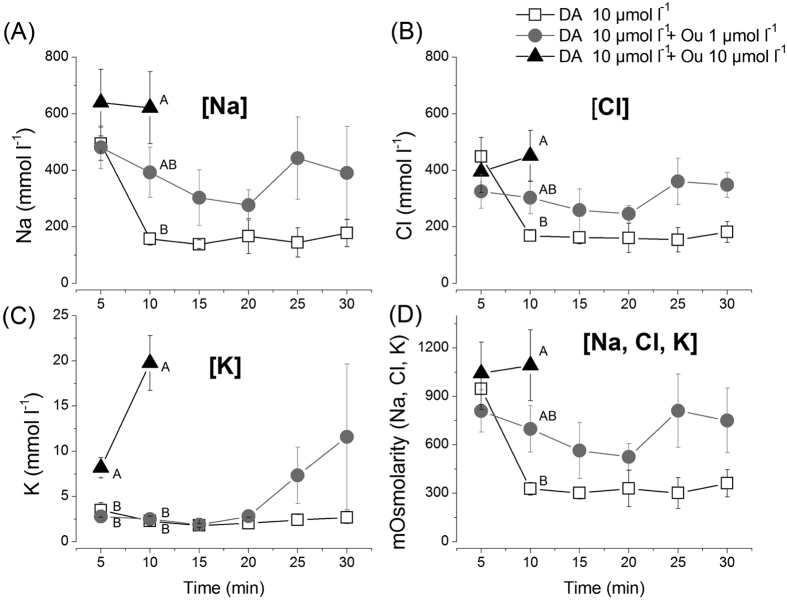
Ion composition and osmotic concentration of secreted saliva from isolated salivary glands. (**A**–**C**) The major three ions’ (Na^+^, Cl^−^, K^+^) secretion patterns over a 30-minute time period following treatments. (**D**) The pattern of total osmotic concentration of three major ions (Na^+^, Cl^−^, and K^+^) in the saliva secreted over a 30 minute time period. SEM/EDS was used to analyze each ion concentration from each time point. Standard curves for each ion (Na^+^, Cl^−^, and K^+^) were generated with NaCl and KCl (see Supplementary Fig. S4). The symbols indicate averages with standard error of the mean (s.e.m.) of three replicates. The data were analyzed by an ANOVA-Tukey-Kramer HSD test (p = 0.05). Different letters indicate significant differences.

The DA or pilocarpine-induced salivary secretion from partially fed *I. scapularis* females were 461–484 mOsmol/L (n = 2–4), hyposmotic to the hemolymph (557 mOsmol/L) at the same feeding stage (Fig. 5). Saliva induced by pilocarpine (10 mg ml^−1^) was 484 ± 23 mOsmol/L (n = 3; Fig. 5). DA-induced saliva from salivary gland bathed in Hank’s saline (~387 mOsmol/L) *in vitro* was 461±27 mOsmol/L (n = 2; Fig. 5). The hyposmotic salivary secretion in *I. scapularis* was similar to that of partially fed *D. andersoni*^14^. However, in partially fed *I. ricinus,* the saliva was hyperosmotic^35^. We speculate that the duration of saliva collection in different experiments may have caused this discrepancy, because we found that DA-induced secretion collected during the first 5 min was highly hyperosmotic (~946 mOsmol/L) with subsequently decreased osmolarity of the saliva (Fig. 6D).

Salivary secretions collected every 5 mins during the course of the modified Ramsey’s assay were subjected to measurement of their ion compositions. The initial analysis of saliva using scanning electron microscopy/energy dispersive X-ray spectroscopy (SEM-EDS) identified concentrations of three major ions: Na^+^, Cl^−^, and K^+^. Sodium-rich saliva has been described in a number of tick species, including *A. americanum*^34^ and *D. andersoni*^14^. When analyzing changes in the osmotic pressure and ion composition of saliva over the 30 min duration following dopamine treatment, an interesting observation was that the DA-only treatment induced high osmolar salivary secretion (946 mOsmol/L) in the first 5 min, but lowered afterward. The saliva secreted in the first 5 min includes significant portion of saliva that is already contained within the salivary ducts and the acini lumen at the equilibrium and is expelled as a result of a DA-induced influx of water/solutes into the acini. Therefore, the saliva at the equilibrium state is likely highly hyperosmolar, but becomes diluted by the subsequent influx of hyposmotic saliva resulting from dopamine treatment.

Ouabain treatments resulting in hyperosmolar dopamine-induced salivary secretion were surprising. The one μmol l^−1^ ouabain treatment showed a mild inhibitory activity on the total volume of salivary secretion (Fig. 3C), but resulted in 1.5-fold higher osmotic salivary secretion than only dopamine-induced salivary secretion (Fig. 5). Also, the concentration of the three major ions (Na^+^, Cl^−^, and K^+^) was higher in combinatory treatment than that in dopamine-only treatment (Fig. 6). Na^+^ and Cl^−^ concentrations were coupled for their fluctuating pattern, while K^+^ exhibited an increase in the last 10 min of the 30 min observation period. At 10 μmol l^−1^, ouabain, dopamine-induced salivation was significantly reduced (Fig. 3), saliva collection was possible only for the first two 5 min intervals. These samples were highly rich in Na^+^ and Cl^−^. The K^+^ concentration was 8.5-fold higher than that in the dopamine-only treatment at the same time interval (Fig. 6).

Current results suggest two opposite physiological roles of Na/K-ATPase: ion and water influx into the lumen of type III (and likely also in type II) acini forming the primary saliva, as was shown by the volume increase in type III acini in the previous section, and the reabsorption present in type I acini located in the proximal region of the main duct (see more about in the last section Absorptive function of type I acini). Although the experimental results fit well in the general outline of the model, there are a number of questions arising.

The dramatic increases in ion concentrations, accompanied by a slight reduction in dopamine-induced salivary secretion volume, like those noted in the 1 μmol l^−1^ ouabain treatment, demands further explanation for the mechanism disrupting the balance between ion/water secretion and resorption. If similar levels of inhibition for Na/K-ATPase occurred in both type I and type III acini, the saliva should have similar ionic concentrations to that of the saliva induced by dopamine alone. A possible explanation for the disrupted ion balance could be that the Na/K-ATPases in type I acini vs. those in type III acini exhibit differing sensitivities to ouabain. Thus, at the lower dose, the resorption function of type I acini was inhibited, while partial secretory function of type III acini remained for the production of the hyperosmolar primary salivary secretion. Both secretion and resorption functions were almost completely blocked by the higher dose (10 μmol l^−1^) of ouabain. The different ouabain sensitivities could be attributed to possible differences in accessibility of the targets by ouabain (note the different localizations of immunoreactivity in Fig. 2), either due to different cellular subunit composition, or to the possibility that different isoforms of Na/K-ATPase possess different posttranslational modifications.

Finally, what could be the contribution of Na/K-ATPase to the dopamine-mediated hyposmotic salivary secretion? Based on the fact that ouabain at 10 μmol l^−1^ almost completely abolished salivary secretion, the ouabain sensitive Na/K-ATPase must be the major component in dopamine’s downstream physiology. The saliva present in the SG prior to dopamine treatment appeared to already be highly hyperosmotic, as inferred from the high osmolarity of saliva secreted in the first 5 min following dopamine treatment (Fig. 6). In this case, activation of Na/K-ATPase to form electrochemical gradients may not be necessary for fluid transport across epithelial cell layers, unless Na/K-ATPase is only functioning for the production of a local osmotic gradient in a subcellular region, such as the labyrinth-like structure present in the epithelial cells. Although significant direct contributions of Na/K-ATPase on the electrochemical gradient may occur, based on the high K^+^ concentrations in the 10 μmol l^−1^ ouabain treatment, Na/K-ATPase might be involved in dopamine-mediated activation of water channels (aquaporins) as a parallel mechanism in the downstream dopamine pathway, which is also inhibited by ouabain. Changes in ionic strengths, whether directly or indirectly mediated by Na/K-ATPase activity in the microdomain, would affect aquaporin activity, just as changes in pH have been shown to control aquoaporin^36^. Regulation of pH in subcellular organelles by Na/K-ATPase has also been reported^37^.

Na/K-ATPase located in the infolding of apical membranes of epithelial cells in type III acini does not appear to be a common phenomenon, while Na/K-ATPase localization in the basolateral regions for resorptive systems (type I acini) is well documented for the epithelial cells of the mammalian kidney^38^. In mammalian retinal pigment epithelium cells, apically located Na/K-ATPase is known to be important for pH control by coupling with Na^+^/HCO3^−^ 39. In addition, the model for insect salivary glands (American cockroach) includes apical Na/K-ATPase in an acinar cell^40^, named the p-cell (peripheral cell), functioning for primary saliva production, and basolateral Na/K-ATPase in an duct cells, functioning for sodium-resorption^40^,^41^.

The electrochemical gradient formed by the efflux of 3Na^+^ towards the lumen and the counter flow of 2K^+^ in type III acini must be coupled with other machineries for the epithelial physiology: extrusion of K^+^ and basolateral influx of Na^+^ in the epithelial cells, and the luminal influx of Cl^−^ and water through transcellular and paracellular routes. Similarly, the resorption function of Na/K-ATPase located on the basolateral infolding of type I acini is likely associated with similar types of machinery, but for the opposite directional flows.

It is worth noting a major difference between Na/K-ATPases in type I and type III acini; the former seems to be dopamine-independent, while the latter is controlled by dopamine. Indeed, a previous study for the localization of dopamine receptors D1 and InvD1L did not find immunoreactivity in the type I acini^24^,^25^, supporting the possibility of dopamine-independent Na/K-ATPase actions.

### Neither DA nor ouabain influence protein content of the saliva

**Figure 7:**
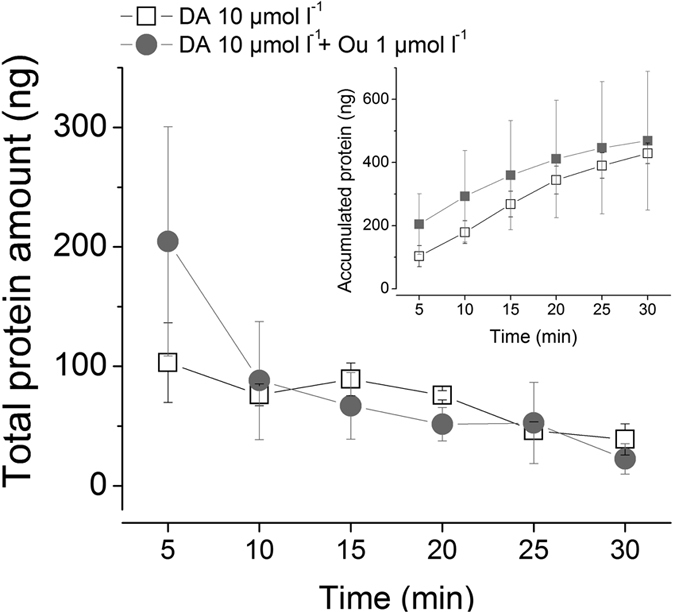
Protein quantification of secreted saliva. Protein secretion over a 30-minute time period following treatments: dopamine (10 μmol l^−1^) alone (empty box) and dopamine (10 μmol l^−1^) with ouabain (1 μmol l^−1^) (grey circle). Inset graph, accumulated protein amounts over 30 minutes after treatments. The data are the averages with standard error of mean (s.e.m.) for at least three biological replicates. Values were plotted after conversion by applying value ratio between CBQCA analysis and TapeStation analysis.

We expanded our scope to examine the effects of dopamine and ouabain on the secretion of proteins in the saliva. We did not find any increased protein secretion from secreted saliva obtained in 5 min interval salivary secretion in modified Ramsay’s assay. Basal protein secretion levels of ~300 μg mL^−1^ protein per 5 min were observed over the 30 min observation period and, like the case of ion concentration, the saliva secreted in the first 5 min contained high amounts of protein (103 ng and 204 ng, respectively) (Fig. 7), which is likely the salivary contents present in the salivary gland prior to dopamine-induced salivation. Therefore, we conclude that neither dopamine nor ouabain affected the basal protein secretion level up to 30 mins after the pharmacological treatments. Our result did not support the earlier model proposed by Sauer’s group^20^. In this model, they proposed a sequential activation of dopamine for basolateral secretion of prostaglandin E_2_ (PGE_2_), which is then responsible for triggering the secretion of salivary proteins, including anticoagulant components^20^,^42^,^43^. Based on this model, increased protein secretion is expected to coincide with increased saliva volume (10 and 15 min after dopamine treatment), which was not observed in our study. We were also unable to find any visible responses of salivary gland acini to PGE_2_ (100 μmol l^−1^) treatment, including vesicular dynamics of type II granular cells (Kim and Park, unpublished data). Further investigation is needed to test the possibility that the dopamine-signaling pathway is responsible for maintaining basal levels (~300 μg mL^−1^ 5 min^−1^) of protein secretion.

Over the course of this study, protein bands obtained during protein quantification by Agilent system provided interesting observation unexpectedly. First, we found that the protein amounts of pilocarpine vs. dopamine inductions were not significantly different on the Agilent 2200 TapeStation, supporting that the muscarinic acetylcholine synapse in the central nervous system and SG autocrine dopamine are in the same pathway^44^. Second, we found high variations among samples (pools of two individuals) in the protein band patterns of the saliva from approximately similar feeding stages of the same batch of ticks (Supplementary Fig. S3). A previous study with *R. appendiculatus* also found high variations in the protein band patterns by pooling multiple groups of five individuals^45^.

### Absorptive function of the type I acini

**Figure 8:**
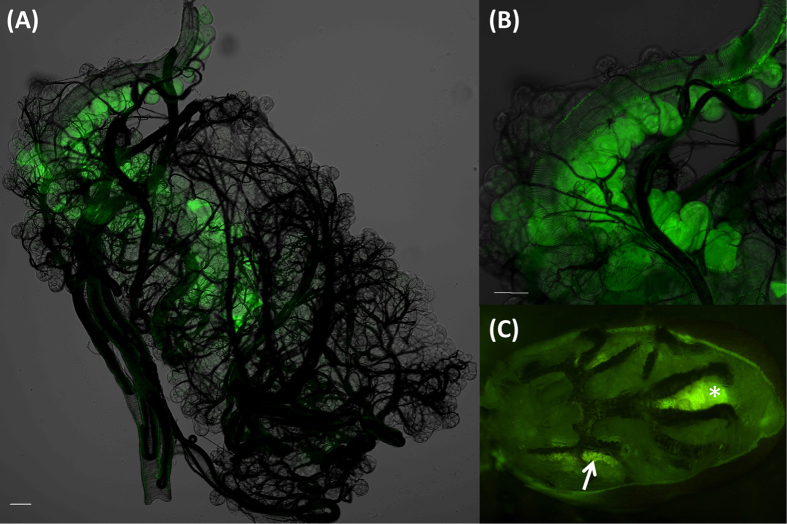
Absorptive function of type I acini shown by absorption of Rhodamine 123. Ticks were dissected to trace the fluorescence after the desiccated tick was offered a drop of water containing Rhodamine 123 (1 mmol l^−1^) for ~5 hr, (**A**) Intact salivary glands of unfed tick. (**B**) Magnified view of type I acini. (**C**) Overview of fluorescence from whole unfed tick body after the tergum was removed. Arrow in (**C**) indicates the salivary glands. Asterisk indicates hindgut and rectal sac that is autofluorescent (See Supplementary Fig. S6). Scale bars equal 50 μm.

Since the combined treatments of ouabain and dopamine led to an increased ion concentration, suggesting the resorptive function of salivary glands for ions and water, we designed an experiment to test whether salivary glands might have an absorptive function. Dehydrated ticks were fed with water containing Rhodamine123 to identify the route of water absorption. We found that type I acini cell bodies were the only site where Rhodamine123 was taken up (4/7 individuals) in the salivary glands (Fig. 8), while some individuals accumulated Rhodamine123 in both the salivary glands and midgut (3/7) (Fig. 8C and see Supplementary Fig. S6). Accumulation of Rhodamine123, which is a commonly used tracer dye for visualizing membrane transport^46^,^47^, in the cells of type I acini only indicates the presence of an absorptive function in those cells. Since dissection was done immediately after the drinking, which was detected by reduced volume of the Rhodamine123, we believe that the Rhodamine123 in type I acini is in the direct absorptive passage. This result obtained in unfed ticks is likely applicable to the feeding stage for the resorptive function of type I salivary acini.

### Conclusion

This study revealed a number of unique features of tick salivary glands

- We propose that the formation of primary saliva in type III and likely also in type II acini is followed by resorption of ions and water in the proximally located type I acini. Filtering functions of excretory organs and the formation of primary excretion, followed by resorption, are commonly found in the renal organs (salivary glands^41^,^48^,^49^ and Malpighian tubules^50^,^51^) of other arthropods.
- We propose that a major downstream physiology in the dopamine-mediated salivary secretion pathway is the action of Na/K-ATPase in the epithelial cells of type III (and likely also in type II) acini for sodium-rich primary saliva formation, while Na/K-ATPase in type I acini is dopamine-independent for its resorptive function.
- The locations of Na/K-ATPase expression, revealed via immunohistochemistry, indicate that the Na/K-ATPase in type I acini is located in the basolateral infoldings, implying its likely role in Na^+^ resorption according to the epithelial model. However, the Na/K-ATPase in type III acini is located in the apical labyrinth-like infolding, implying a Na^+^ secretion function. We hypothesize that these differing functions of Na/K-ATPase at different locations are similar to the proposed model for salivary secretion of cockroaches^41^. The observed localizations and functions (i.e., hyposmotic salivary secretion) in this study suggest that Na/K-ATPase functions, not only for local electrochemical gradient formation, but also potentially for the activation of the water channel, aquaporin.
- Evidence for an absorptive function of type I acini, which has been suspected in previous studies, is provided via the fluorescent dye experiment in this study.

## Methods

### Ticks and salivary glands

Blacklegged ticks (*Ixodes scapularis*) were purchased from the tick rearing center at Oklahoma State University. Partially engorged female ticks (the weight 12–21 mg, 4–5 days blood fed) were prepared using a modified artificial feeding system^52^. Salivary glands were dissected from partially engorged female ticks and pre-incubated in Hank’s saline buffer for 1h before experimental treatment. For immunolocalization, salivary glands were immediately fixed after dissection.

### Molecular cloning, manual annotation, and confirmation of isoforms

The sequence data for Na/K-ATPase, ISCW002538, was downloaded from Vectorbase^53^ (https://www.vectorbase.org). We made manual annotations by correcting the ISCW002538 sequence based on its homology with other arthropod Na/K-ATPases and determining the translation initiation site prediction via GeneFinder (www.softberry.com). For experimental verification of the mRNA sequence, cDNA was synthesized from total RNA extracted from two pairs of salivary glands of partially fed female ticks using Direct-zol™ RNA miniprep with TRI-Reagent^®^ (Zymo). Salivary gland total RNA (500 ng) served as the template for cDNA synthesis via SuperScript^®^ III Reverse Transcriptase (Invitrogen). Using New England Biolabs (NEB) *Taq* DNA polymerase with ThermolPol^®^ buffer (NEB), RT-PCR was performed to amplify the 5′ terminal end of Na/K-ATPase, including the 5′ UTR, exon1, exon2, and isoforms -a and -b via different PCR conditions, depending on the regions of the Na/K-ATPase transcript. All primers are provided in Table 1. PCR reactions were conducted in 25 μL volumes, including 10X NEB ThermoPol buffer, 0.1 μM each dNTPs, and 0.4 μM of each primer. PCR programs were as follows: for 5′ terminal of Na/K-ATPase (95 °C for 30 sec, 30 cycles: 95 °C for 30 sec, 61 °C for 30 sec, 68 °C for 90 sec, with a final extension of 5 min at 68 °C); for nested PCR of the 5′ terminal of Na/K-ATPase (95 °C for 30 sec, 35 cycles: 95 °C for 30 sec, 60 °C for 30 sec, 68 °C for 30 sec, with a final extension for 5 min at 68 °C); for isoforms -a and -b (95 °C for 30 sec, 35 cycles: 95 °C for 30 sec, 58 °C for 30 sec, 68° for 30 sec, with a final extension of 5 min at 68 °C). For amplification of the region from exon15 to exon19, a high fidelity enzyme, PrimeSTAR^®^ HS DNA polymerase (TAKARA), was used. PCR products were subsequently purified and cloned into the pGEM^®^-T Easy vector (Promega) and sequenced.

In order to distinguish between isoform-a and isoform-b, PCR products of each isoform were digested with a specific restriction enzyme (BstXI), which digested only isoform-b but not isoform-a (see Supplementary Fig. S1). After restriction enzyme digestion, products were run on a 1% agarose gel for confirmation.

### Phylogenetic analysis

Using MEGA6^54^, a phylogenetic tree was constructed using the neighbor joining method, with 1000 bootstrap replicates (Fig. 1D). The deduced amino acid sequence of *I. scapularis* Na/K-ATPase were analyzed via multiple sequence alignment with other Na/K-ATPase genes of, not only *Homo sapiens,* but also other arthropods: *Metaseiulus occidentali*, *Neoseiulus cucumeris*, *Drosophila melanogaster*, *Tribolium castaneum*, *Apis mellifera*, and *Aedes aegypti*.

### Quantitative RT-PCR

To obtain a stage-specific expression profile of the Na/K-ATPase transcript, total RNA was extracted from three pairs of salivary glands of ticks at each of six different feeding stages (12 hrs, 1 day, 2 days, 3 days, 4 days, and 5 days after feeding) via Direct-zol™ RNA miniprep with TRI-Reagent^®^ (Zymo). Subsequently, the salivary gland total RNAs (500ng) served as templates for cDNA synthesis using SuperScript^®^ III Reverse Transcriptase (Invitrogen). qRT-PCR experiments were performed using SYBR Select Master Mix (Applied Biosystems) in a CFX Connect (Bio-Rad) thermal cycler on a 96-well plate with optical sealing tape. All reactions were carried out in duplicate, in 10 μl volumes. Na/K-ATPase quantification was performed using primers targeting a 98-bp fragment of the Na/K-ATPase transcript (F: 5′-AGACATGCTGCCTGCTATTTC-3′; R: 5′CGTTCACCAGTTTGTCCTTC-3′), while a 174-bp fragment (Primers: F: 5′-GGTGAAGAAGATTGTCAAGCAGAG-3′; R: 5′-TGAAGCCAGCAGGGTAGTTTG-3′) of the *I. scapularis* gene, Ribosomal Protein S4 (RPS4, GenBank Accession No. DQ066214), served as an internal reference gene^55^. Quantification of Na/K-ATPase expression levels was calculated via the ΔΔCt method. Transcript levels are expressed as the fold difference, relative to the expression levels exhibited by salivary glands from the 12 hr time point, where error bars represent the experimental variation among technical replicates.

### Immunolocalization of Na/K-ATPase

To localize *I. scapularis* Na/K-ATPase, a mouse monoclonal antibody (a5) against chicken Na/K-ATPase^56^ was purchased from the Developmental Studies Hybridoma Bank at the University of Iowa, IA, USA. Partially engorged female ticks were dissected in Hank’s saline buffer and then fixed in Bouin’s solution (37% formaldehyde and saturated solution of picric acid 1:3) at 4 °C for 2 days. Fixed salivary glands were washed with PBS containing 1% Triton X-100 (PBST). After incubation with 5% normal goat serum (Jackson ImmunoResearch) for 20 minutes, the tissue was incubated with the a5 antibody at 4 °C for 2 days. Following primary antibody incubation, tissues were washed with PBST and subsequently incubated with a goat-anti-mouse IgG antibody, conjugated with Alexa Fluor 488 (Molecular Probes), overnight at 4 °C. Tissues were then washed with PBST, incubated in 300 nM 4′,6′-diamino-2-phenylindole (DAPI, Sigma) for 5 minutes, and then mounted in glycerol. Images were captured with a confocal microscope (Zeiss LSM 700).

### Assay measuring the responses of individual type III acinus

All details were described in a previous study^26^. Briefly, dissected salivary glands were pre-incubated in Hank’s saline buffer for ~1 hr. Pharmacological agents were then applied into either individual acinus. Dynamic changes of acini were video recorded and analyzed by measuring the radius of individual acini. The acini volume change and percentage of pumping/gating were analyzed every 2 minutes for a total of 30 min.

### Modified Ramsay’s assay and collection of salivary secretion

All details were described in a previous study^26^. Briefly, salivary glands were placed in a droplet of Hank’s saline on a petri dish coated with Sylgard^®^ (World Precision Instrument) after an hour pre-incubation. The main duct was pulled out from the Hank’s saline droplet crossing over a narrow high vacuum grease dam (0.5 – 1 mm) and immobilised on the Sylgard^®^ petri dish surface. To collect secreted saliva, heavy mineral oil covered both the Hank’s saline and the main salivary gland duct. A micro-injector (Nanoliter 2000, World Precision Instrument) controlled by a micro-syringe pump controller (Micro4, World Precision Instrument) was used for withdrawing the secreted saliva, and the volume withdrawn was recorded in every 5 min for 30 min. For dopamine assay, we measured secreted saliva for 30 min after drug treatment. For ouabain assay, salivary glands were pre-incubated in ouabain for 25 min, and then dopamine was administered to salivary glands in the presence of ouabain. Secreted saliva was spotted into a layer of heavy mineral oil on a slide glass and stored at −80 °C for the further analysis. Dopamine (Sigma) and ouabain (LC Laboratory) were purchased.

### Protein quantification of secreted saliva

We have established a fluorescent dye-based method for protein quantification from 2 nL samples collected from the modified Ramsay’s assay. To quantify protein amounts in secreted saliva, a modified CBQCA protein quantification method (Invitrogen) was adopted. A Nanoliter2000 (WPI) was used to spot drops of both protein standards (bovine serum albumin, BSA) and secreted saliva onto a slide glass, which was covered by heavy mineral oil to prevent evaporation. Next, the same volume of reaction mixture (0.1M sodium borate (2): KCN (1): CBQCA (2)) was added into the saliva drops. Following a 1-hour incubation of reactions in the dark, images of each nanoliter drop were captured using a CoolSnap ES2 CCD camera (Photometrics). Two factors were used for the calibration of protein amounts: Serial dilutions of BSA (0, 0.5, 1, 2, and 4 mg ml^−1^; see Supplementary Fig. S2) and tick saliva from a partially fed tick whose secretion was induced by pilocarpine or dopamine. A protein standard curve was obtained from the tested concentrations of BSA. Since the tick saliva contains non-protein components, causing over estimation of protein amounts in the tick saliva, we corrected the estimated quantity using the quantity measured in the Agilent 2200 TapeStation that requires a 2 μL sample of saliva (Agilent Technologies, see Supplementary Fig. S3). Samples used for the calibration consisted of saliva collected following the injection of pilocarpine (10 mg ml^−1^) and dopamine (10 μmol l^−1^) into partially fed females (5-days after the onset of feeding, ~21 mg in the weight). Saliva samples were pooled from two individuals, totaling six pools that were tested both in the Agilent 2200 TapeStation and in the fluorescent dye-based method, and the calibration was made.

### Measurement of major ions (Na^+^, Cl^−^, and K^+^) in secreted saliva

Secreted saliva was analyzed to obtain elemental composition and concentrations via scanning electron microscopy (SEM)/energy-dispersive X-ray spectroscopy (EDS) with a silicon drift detector (SDD), which allowed us to overcome the limitation of small volumes of saliva. Using a Nanoliter2000 (WPI) 2 nl of saliva was spotted onto the silicon wafer grid substrate (Pelcotec^TM^) (see Supplementary Fig S5), which was dried and kept at room temperature until analysis. A Nova NanoSEM 430 (FEI Company) equipped with EDS (Oxford Instruments) was used to image and analyze each dried saliva spot, and INCA software version 4.15 (Oxford Instruments) provided elemental values as weight percentages and atomic percentages. The value of the substrate silicon was used as an internal control or background. To get a standard curve for each element (Na^+^, Cl^−^, and K^+^), NaCl (Fisher Scientific) and KCl (Fisher Scientific) were spotted onto the silicon substrate with differing concentrations: NaCl (10, 50, 100, 200, 400, 600 and 800 mmol l^−1^) and KCl (2, 4, 8, 16, 32, 64, and 100 mmol l^−1^). The standard curves for each ion were generated based on atomic % values of each concentration (see Supplementary Fig S4 and Supplementary Fig. S5). The osmotic concentrations of secreted saliva were also measured by osmometer (5100B Vapor pressure osmometer, Wescor, Inc.). The differences in the rates of osmotic concentrations between SEM/EDS and osmometer were applied to convert the finalized values for ion osmotic concentrations (Fig. 6).

### Rhodamine123 and water ingestion into dehydrated unfed female ticks

In order to dehydrate unfed female ticks, unfed ticks were placed in an incubator with 25% relative humidity (RH) for 12 hours. Rhodamine123 (Sigma) 1 mmol l^−1^ or water filled 1 μL micropipettes (Drummond) were then placed onto the mouthparts (chelicera) of dehydrated unfed female ticks. Ticks were then placed into rehydration conditions (RH 98%) for 15 min. to 5 hours until the reduction in the volume of Rhodamine123 solution was detected. After ingestion of Rhodamine123 or water, salivary glands were dissected, fixed in 4% formaldehyde at room temperature for 1 hour, washed in PBST (1% Triton X-100), and imaged on a confocal microscope (Zeiss LSM 700).

## Additional Information

**How to cite this article**: Kim, D. *et al.* Multiple functions of Na/K-ATPase in dopamine-induced salivation of the Blacklegged tick, *Ixodes scapularis. Sci. Rep.* **6**, 21047; doi: 10.1038/srep21047 (2016).

## Supplementary Material

Supplementary Information

## Figures and Tables

**Table 1:** Primer information.

Purpose for primers	Primers (5′ to 3′)
5′ terminal forward primer	CTTGAGGTGGCGGTGCAG
5′ terminal reverse primer	GATGGCATACTGTGGAACCATG
5′ terminal nested forward primer	GACGCAGCCCTAGTGCGTG
5′ terminal nested reverse primer	CAATCCACAGTAGTAAGGAGAAC
Exon15-19 specific forward primer	CTGGAATATACCTGCCATAC
Exon15-19 specific reverse primer	GTATGGCAGGTATATTCCAG
Isoform-a specific reverse primer	TCAGCGGGTTCCGGGGCTGA
Isoform-b specific reverse primer	GAAGGGGATCGCGCGGTCTT
qPCR-Na/K-ATPase forward primer	AGACATGCTGCCTGCTATTTC
qPCR-Na/K-ATPase reverse primer	CGTTCACCAGTTTGTCCTTC
qPCR-RPS4 forward primer	GGTGAAGAAGATTGTCAAGCAGAG
qPCR-RPS4 reverse primer	TGAAGCCAGCAGGGTAGTTTG
