# Supplementary material for: Multiple functions of Na/K-ATPase in dopamine-induced salivation of the Blacklegged tick, Ixodes scapularis
Source: Sci Rep. 2016 Feb 10;6:21047. doi: 10.1038/srep21047 (PMC4748274; doi:10.1038/srep21047)

Supplementary information

Multiple functions of Na/K-ATPase in dopamine-induced salivation of the Blacklegged tick, *Ixodes scapularis*

Donghun Kim[1], Joshua Urban[1], Daniel L. Boyle[2], and Yoonseong Park[1*]

[1]Department of Entomology, Kansas State University, 123 Waters Hall, Manhattan, KS 66506, USA, [2]Division of Biology, Microscopy Facility, Kansas State University, Ackert Hall, Manhattan, Kansas 66506, USA

[*]Corresponding author (ypark@ksu.edu)

Supplementary Figure S1. Na/K-ATPase in the salivary glands utilizes mainly exon 17, while low levels of transcripts using exon 16 also exist. Only exon 16 carries the BstXI restriction motif and the BstXI-cut was confirmed by electrophoresis of the exon 16 PCR product and subsequent digestion.

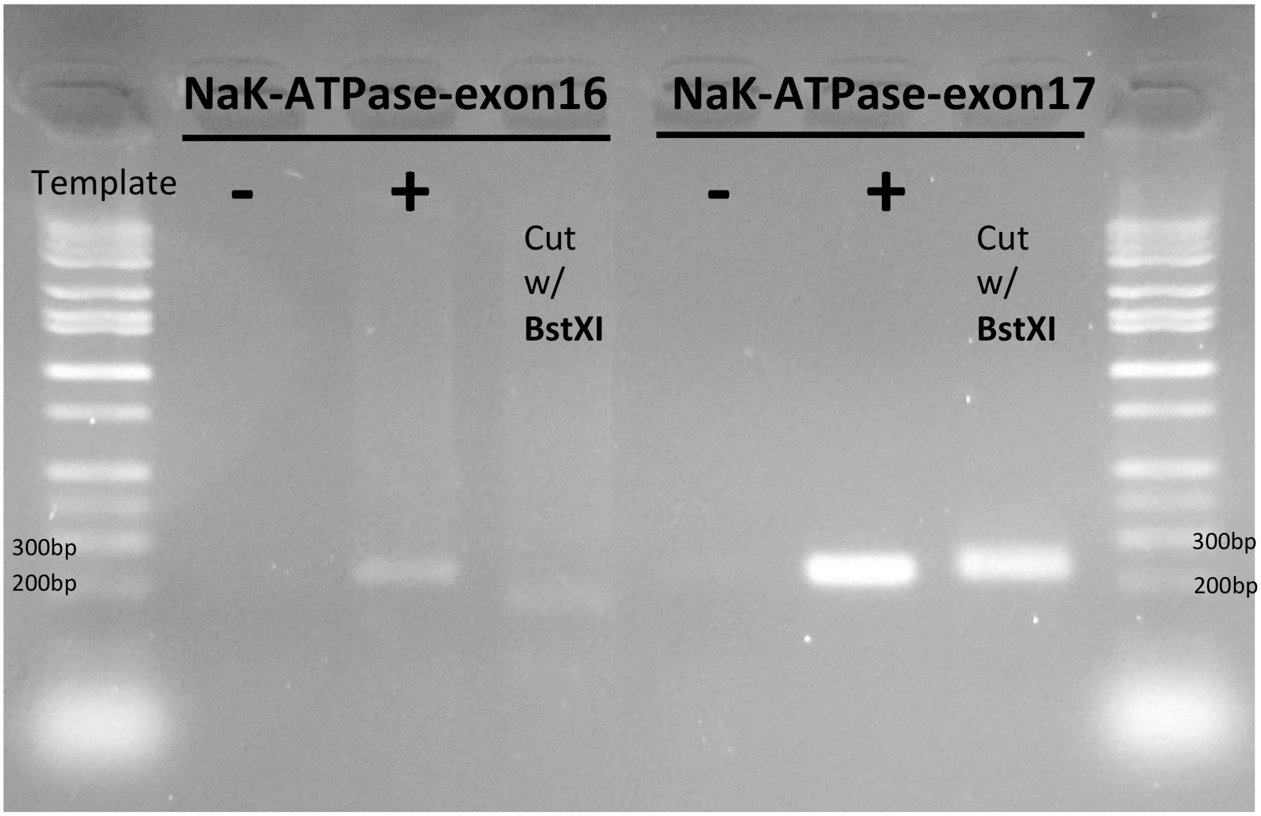

Supplementary Figure S2. Protein quantification using fluorescence-dye based method (CBQCA). Standard curve: bovine serum albumin (BSA, Serial 2-fold dilutions from 0 mg ml^{-1} – 4 mg ml^{-1}) and captured images of fluorescence from each concentration of BSA.

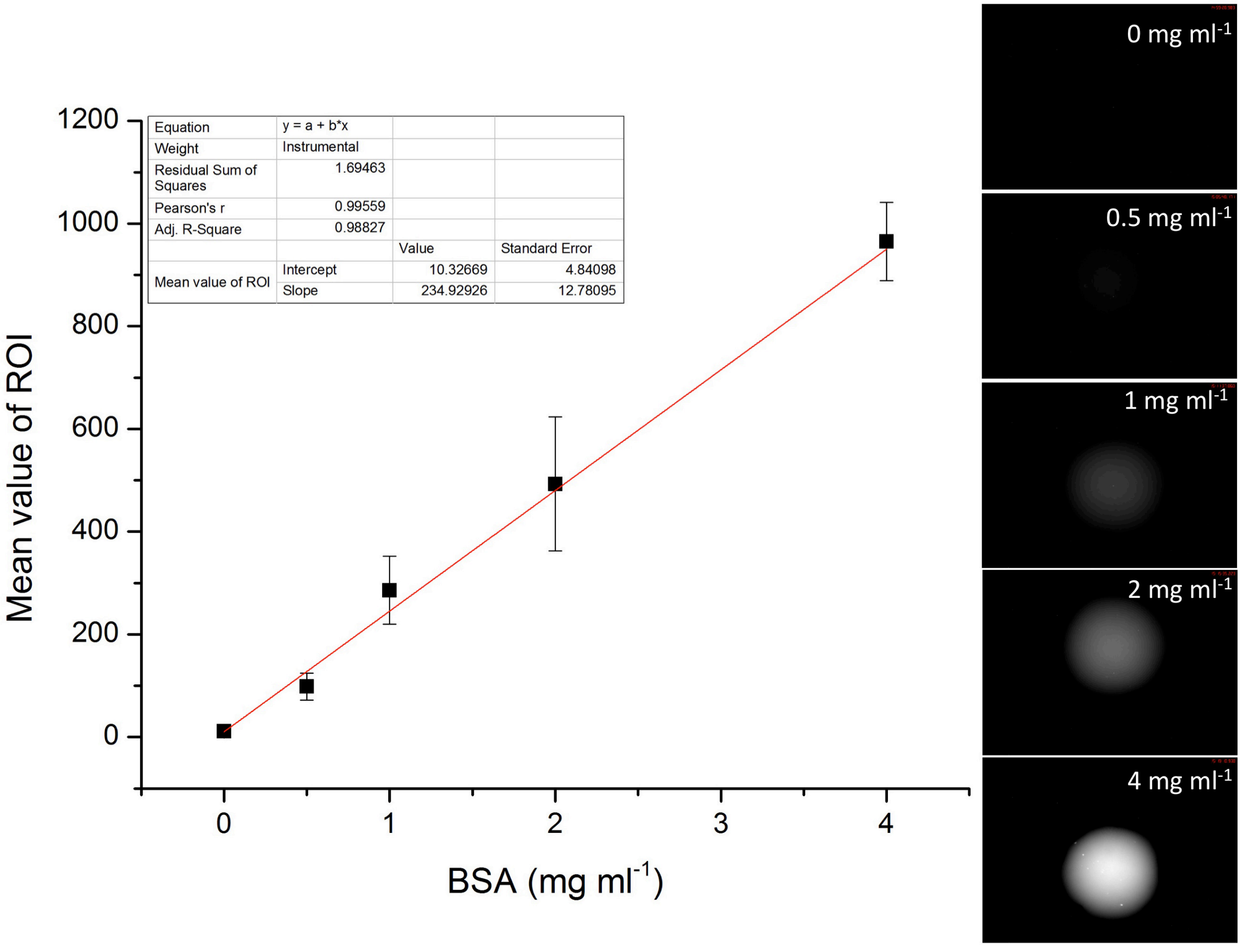

Supplementary Figure S3. Variations in the salivary proteins shown by Agilent 2200 TapeStation. Each lane represents pooled saliva collected from two individuals. A1: P200 ladder. B1-D1: saliva induced by injection of 2µl of pilocarpine (10mg/ml). E1-H1: saliva induced by injection of 2µl of dopamine (10µmol l^{-1}). The band pattern indicated large variations among different individuals, but not between different methods of inducing salivation.

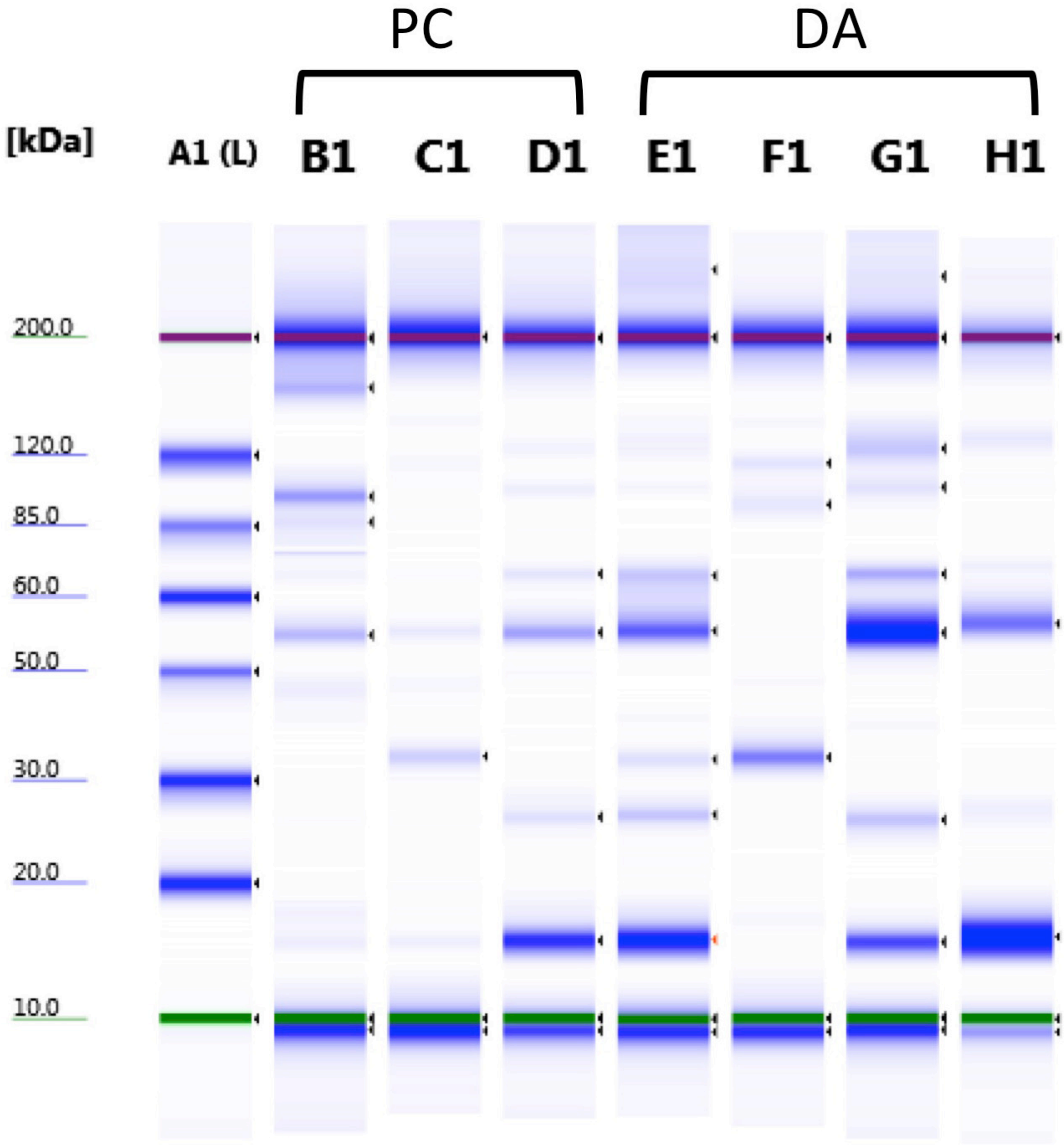

Supplementary Figure S4. Standard curves of major ions analyzed by SEM/EDS. (A) Na^{+}; (B) Cl^{-}; (C) K^{+}.

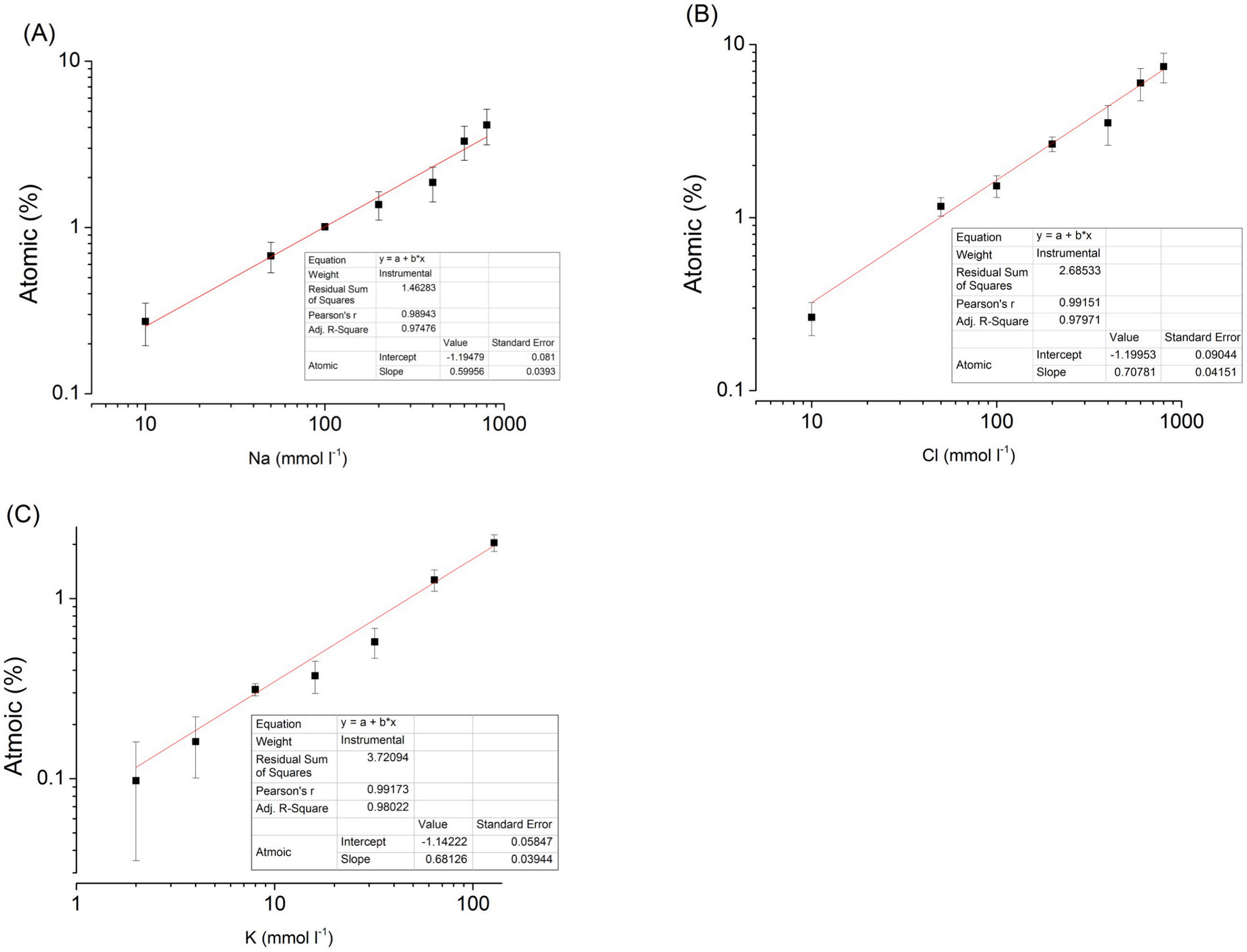

Supplementary Figure S5. SEM images. A Nanoliter2000 (WPI) was used to place a drop (2 nL) of either standard solution or saliva onto the silicon grid. (A) NaCl standard (10mM to 800mM); (B) KCl standard (2mM to 124mM); (C) Representative image of a drop of saliva. The scale bar indicated is 45μm in length.

(A)

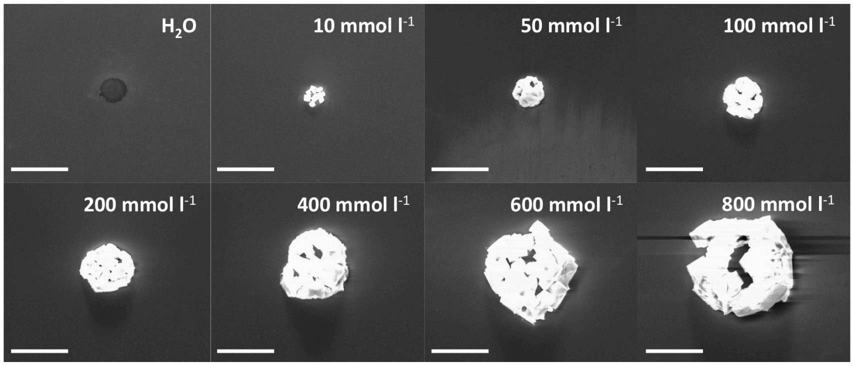

(C)

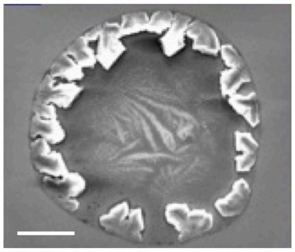

(B)

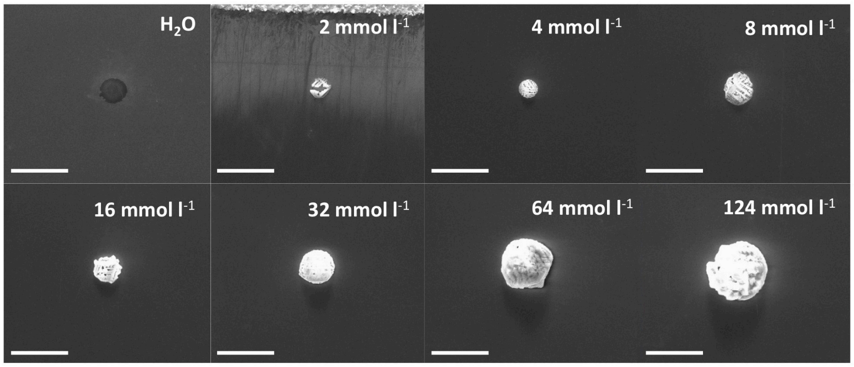

Supplementary Figure S6. Fluorescence observation from water ingested (negative control) unfed tick. Auto-fluorescence was observed from the hindgut and rectal sac of a water ingested tick. (A) Overview of fluorescence from water ingested tick. (B) Lack of fluorescence from intact isolated salivary gland.

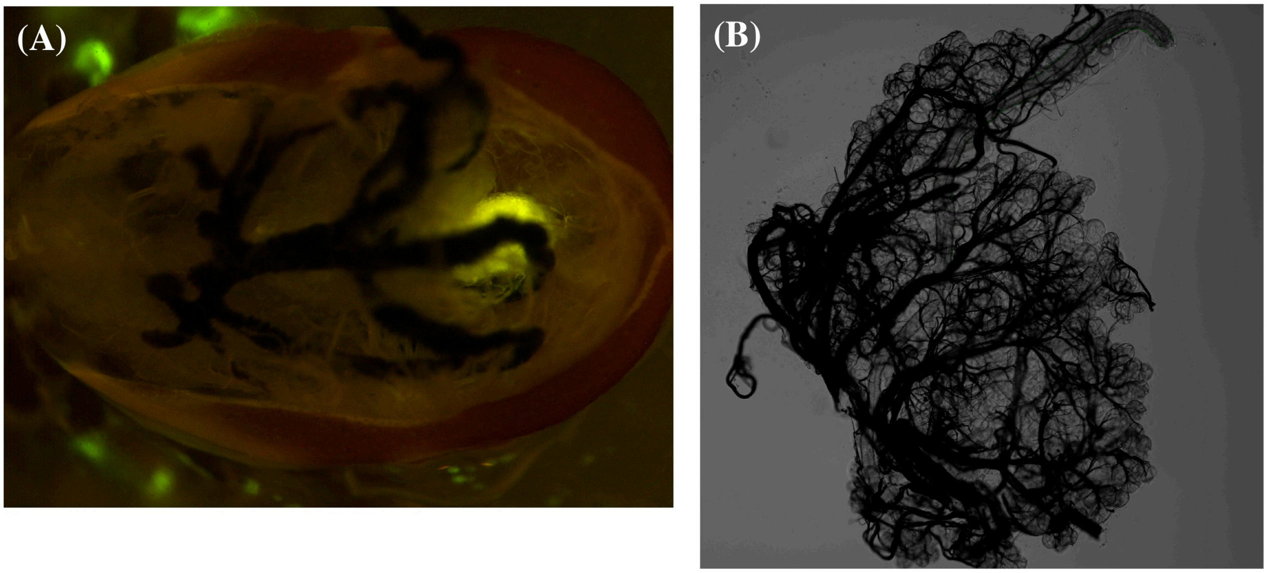

Supplement: Supplementary Information [file srep21047-s1.pdf]
